# Ocular Biometric and Optical Coherence Tomography Parameters in Former Preterm Children: A Cohort Study

**DOI:** 10.1155/2024/2381582

**Published:** 2024-09-30

**Authors:** Marzieh Najjaran, Siamak Zarei-Ghanavati, Hadi Ostadimoghaddam, Abbasali Yekta, Nasser Shoeibi, Armin Hemmati, Mojtaba Abrishami, Saeed Akhlaghi, Mohammed Ziaei

**Affiliations:** ^1^ Department of Optometry School of Paramedical Sciences and Rehabilitation Mashhad University of Medical Sciences, Mashhad, Iran; ^2^ Refractive Errors Research Center Mashhad University of Medical Sciences, Mashhad, Iran; ^3^ Eye Research Center Department of Ophthalmology Mashhad University of Medical Sciences, Mashhad, Iran; ^4^ Ocular Oncology Service Department of Ophthalmology and Visual Sciences University of Toronto, Toronto, Canada; ^5^ Department of Biostatistics School of Health Mashhad University of Medical Sciences, Mashhad, Iran; ^6^ Department of Ophthalmology New Zealand National Eye Centre Faculty of Medical and Health Sciences University of Auckland, Private Bag 92019, Auckland 1142, New Zealand

## Abstract

**Purpose:**

To compare biometric and optical coherence tomography parameters as well as refractive status in preterm children aged 4–8 years with or without retinopathy of prematurity (ROP), and evaluate their correlations with age and gender-matched full-term children.

**Methods:**

Retrospective comparative cohort study of four groups of children. Children with a history of preterm birth, including ROP who received intravitreal bevacizumab (IVB) treatment, children with a history of ROP that regressed without treatment and those with no history of ROP were compared to age and gender-matched full-term children as a control group. Best corrected visual acuity (BCVA), spherical equivalent of refraction (SE), macular and choroidal thickness, as well as biometric parameters was measured.

**Results:**

A total of 120 eyes of 120 children (30 children in each group) were included. There was no significant difference in BCVA, SE, and subjective cylinder between groups (*p*=0.05, *p*=0.3, *p*=0.6, respectively). Axial length was significantly shorter, and the cornea was steeper in both ROP groups than in other groups (*p*=0.001, *p* < 0.001, respectively). The central macular thickness was significantly thicker in the treated, regressed ROP and preterm groups than in full-term children (*p* < 0.001). The gestational age was negatively correlated with macular thickness in both treated and regressed ROP groups (*r* = −0.517; *p*=0.003, *r* = − 0.490; *p*=0.006, respectively).

**Conclusions:**

Children with a history of ROP had a shorter axial length, steeper cornea, and thicker macula that correlated with lower gestational age.

## 1. Introduction

Retinopathy of prematurity (ROP) is a vasoproliferative disease and a significant cause of childhood blindness worldwide. ROP has a high incidence in developing countries and a relatively high prevalence of 23.5% in Iran [1]. Laser photocoagulation as a conventional treatment and intravitreal injection of antivascular endothelial growth factor (anti-VEGF) as a more recent treatment option with favorable structural and developmental outcomes [2, 3], are two main treatment alternatives for ROP.

ROP is associated with several anterior and posterior segment alterations [4, 5]. The underlying pathophysiology of ROP, as well as its treatment modalities [6–8], and the effect of biometrical parameters are postulated to be the main contributing factors for refractive error development in premature children.

Of note, higher rates of myopia have been reported after ROP intervention, as more traditional treatments such as Laser photocoagulation or cryotherapy often disrupts normal ocular growth and development [9, 10]. More recent treatments, however, have been reported to result in less myopia with intravitreal anti-VEGF injections resulting in less myopia compared to laser photocoagulation [7, 11].

Identifying the extent of refractive error as well as macular abnormalities, and biometric differences in children with ROP with or without prior treatment provides valuable information for planning screening and follow-up of these children, particularly during preschool years. Moreover, with the advent of noncontact and noninvasive ocular imaging instruments such as optical coherence tomography (OCT) and biometry a much better understanding of retinal abnormality and biometric parameters differences with quantitative and qualitative measurements is possible in such children.

Few studies have examined structural changes in children with a history of ROP using OCT and biometric parameters [7, 11]. Furthermore, there is a lack of studies reporting on the long-term outcomes of children who have received intravitreal bevacizumab (IVB) [7]. There have been also reports of the crucial need for long-term follow-ups for IVB-treated children due to the late retinal abnormalities even years after treatment [12].

This study sought to assess and compare biometric and OCT measurements as well as refractive status of preterm groups of children with ROP children who received IVB treatment, ROP children who regressed without treatment and preterm children with no history of ROP, with age and gender-matched full-term children as a control group.

## 2. Methods

This retrospective comparative cohort study was conducted at Khatam eye hospital. The study adhered to the Declaration of Helsinki and was approved by the Mashhad University of Medical Sciences Ethics Committee (Code Number: IR.MUMS.REC.1399.613). Informed written consent was obtained from all participants parents/guardians before participation in this study. The medical records of all children receiving IVB treatment during 2013–2017 for ROP, aged 4–8 years at the time of data gathering, were examined.

A sample size calculation was performed using an alpha 0.05 and beta 0.2 parameters according to the study by Wu et al. [13]. This produced a minimum sample size of 30 for each group. They were age and gender-matched with three other groups of children (n: 30 each groups), including ROP children who regressed without treatment, preterm children with no history of ROP who randomly selected through chart review and full-term children with a gestational age (GA) ≥37 weeks and birth weight (BW) ≥2500 g who came for routine eye screening examination served as a control group. Informed written consent was obtained from parents or legal guardians of each child from all four groups before participation.

The inclusion criteria for preterm birth groups were gestational age (GA) ≤32 weeks or birthweight (BW) <2000 grams (g) with adequate follow-up until complete retinal vascularization or regression was achieved with a normal fundus appearance and good cooperation for eye imaging. The IVB-treated group had a history of single treatment with injection of bevacizumab 0.625 mg (0.025 mL) (Avastin; Genentech, South San Francisco, California, USA) by the same surgeon. Exclusion criteria included a history of laser treatment, other ocular diseases, e.g., cataract or glaucoma, ROP sequelae such as macular fold, dragging, or retinal detachment, as well cerebral or congenital defect or significant systemic comorbidity.

### 2.1. Ophthalmic Examinations

GA, BW, delivery type, stage, and zone of ROP were recorded. All children underwent a complete ophthalmic examination by a retinal specialist (A.H.). Ophthalmic investigations were completed by the optometrist in order as follows: Uncorrected visual acuity (UCVA) and best-corrected visual acuity (BCVA) were assessed using a Snellen chart and converted to logMAR (log of the minimum angle of resolution). Objective refraction was assessed with an auto kerato-refractometer (Topcon KR-1; Topcon Corporation, Tokyo, Japan), followed by retinoscopy and subjective refraction to achieve optimal results.

Biometric imaging, including axial length (AL), central corneal thickness (CCT), keratometry readings (K1, K2, mean K), lens thickness (LT), anterior chamber depth (ACD), and horizontal white-to-white (WTW), were measured using the IOLMaster 700 (Carl Zeiss, Jena, Germany) with newer swept-source optical coherence tomography technology and the mean of three good quality measurements were calculated.

OCT imaging was obtained using the Optopol spectral domain OCT system (Revo NX, Optopol Technology, Zawiercie, Poland). This instrument has a superluminescent laser diode (830 nm) as a light source. It completes 110,000 scans per second with an axial resolution of 5 *μ*m, transverse resolution of 12 *μ*m, and a single scan depth of 2.4 mm. All measurements were taken in the same dark room with a nondilated pupil.

The macular thickness data were automatically calculated into nine regions of the Early Treatment Diabetic Retinopathy Study (ETDRS). A central foveal ring with a 1 mm diameter and the inner macular ring with a 3 mm diameter and the outer macular ring with a 6 mm diameter and four quadrants, including superior, inferior, nasal, and temporal were analyzed. Ganglion cell layer + Inner plexiform layer (GCL + IPL) thickness was automatically calculated in six sectors, including three in the superior region (superior, superonasal, and superotemporal) and three in the inferior region (inferior, inferonasal, and inferotemporal).

Choroidal thickness was measured manually using the software tool incorporated into the device, perpendicularly from the outer edge of the retinal pigment epithelium to the choroid-sclera boundary at the fovea by two trained and independent examiners and an average of three measurements was used for further analysis.

Intraocular pressure (IOP) was recorded by iCare IC200 (Icare Finland Oy) tonometer. Six consecutive measurements were taken using a disposable probe from a distance of about 5 mm to the cornea without the need for topical anesthesia. The mean of the best four measurements was used for analysis.

The final ophthalmic investigation was cycloplegic refraction which was completed 30 min after installation of three tropicamide 1% eye drops with five minutes apart, followed by measurement using an auto kerato-refractometer and retinoscopy. Cyclorefraction data was recorded in spherical equivalent (SE).

### 2.2. Statistical Analysis

Data were analyzed using SPSS version 23.0 (SPSS, Chicago, IL, USA). Data were expressed as mean ± standard deviation or median, interquartile range (IQR), as appropriate. The normality of the values was analyzed using the Kolmogorov–Smirnov test. The chi-square test (*χ*^2^) was used for categorical variables. The ANOVA test for normally distributed measurements or Kruskal–Wallis test for non-normally distributed variables were used to compare numerical values between groups. For intergroup post hoc comparisons of ANOVA and Kruskal–Wallis tests, Tukey's (HSD) or Mann–Whitney *U* Test with Bonferroni Adjustment were conducted, respectively. Multiple linear regressions were performed for the multivariable analyses of macular thickness. Spearman correlation coefficient was used to evaluate the correlation between variables. Only data from right eyes were included in the analysis. A *p* < 0.05 was considered significant.

## 3. Results

### 3.1. Patient Characteristics

A total of 120 eyes from 120 children in four groups were included in the study.  Table 1 summarizes the baseline characteristics of children. Half the children were male. The mean ± SD age of children was 6.63 ± 1.25 years (range 4 to 8 years). The treated ROP group had significantly lower median GA as well as lower BW than other groups (*p* < 0.001) except the regressed group. There was no significant difference in mean BCVA, (*p*=0.05), nor SE between the groups (*p*=0.3). IOP was significantly higher only in the regressed ROP group compared to the preterm group in this study (*P*=0.012).

### 3.2. Biometric Components Analysis

Biometric data parameters are demonstrated in Table 2. Post hoc analysis showed that both treated and regressed groups had significantly shorter AL than the control group (*p*=0.002,  *p*=0.002, respectively). Preterm children had significantly shallower ACD than the control group (*p*=0.045) and both treated and regressed ROP groups had marginally shallower ACD than the control group (*p*=0.05 and *p*=0.057, respectively). Both treated and regressed groups had higher K1 (flat) than preterm and control (*p* < 0.001, *p* < 0.001, respectively). The treated group also had significantly higher K2 (steep) than both the preterm and control group (*p*=0.002 and *p* < 0.001, respectively). The regressed group had higher K2 than the control group (*p*=0.003). Both treated and regressed groups had higher mean K than the control group (*p* < 0.001, *p* < 0.001, respectively). Treated, regressed and preterm children had significantly smaller WTW than full-term (*p* < 0.001, *p* < 0.001, and *p*=0.015, respectively).

In the regressed group, there was a positive correlation between AL with BW (*p*=0.011*r* = 0.456), ACD with BW (*p*=0.009*r* = 0.470), and CCT with GA (*p*=0.005*r* = 0.50). The treated group had a negative correlation between AL and SE (*p*=0.039*r* = − 0.379). The full-term group had a positive correlation between average keratometry with SE (*p* < 0.001*r* = 0.630).

### 3.3. OCT Findings

The retinal thickness analysis is demonstrated in Table 3. Post hoc analysis showed that central macular thickness in treated, regressed and preterm groups were significantly thicker than the control group (*p* < 0.001,  *p* < 0.001 and *p*=0.034, respectively). The treated group also had a significantly thicker central macula than the preterm group (*p*=0.004). However, there was no significant difference in central macular thickness between treated and regressed ROP groups (*p*=0.176).

In analysis of the 3 mm diameter of macular thickness, the treated group was thinner in the superior region than the controls (*p*=0.036). There was no other significant difference for 6 mm diameter between groups. A negative correlation was shown between GA and central macular thickness in the treated and regressed groups (*r* = −0.517; *p*=0.003, *r* = −0.490; *p*=0.006, respectively). There was no correlation between central macular thickness with BW and BCVA. Multiple regression analysis considering different parameters, including GA, BW, biometric components and SE, demonstrated that a lower GA was correlated with a thicker central macular region (*ß* = −3.65; 95% CI of *ß*, −6.67 to −0.64; *p*=0.018). There was no significant difference in choroidal thickness between groups (*p*=0.9). A negative correlation was found between choroidal thickness and BCVA in the treated group (*r* = −0.425; *p*=0.019).

**Figure 1:**
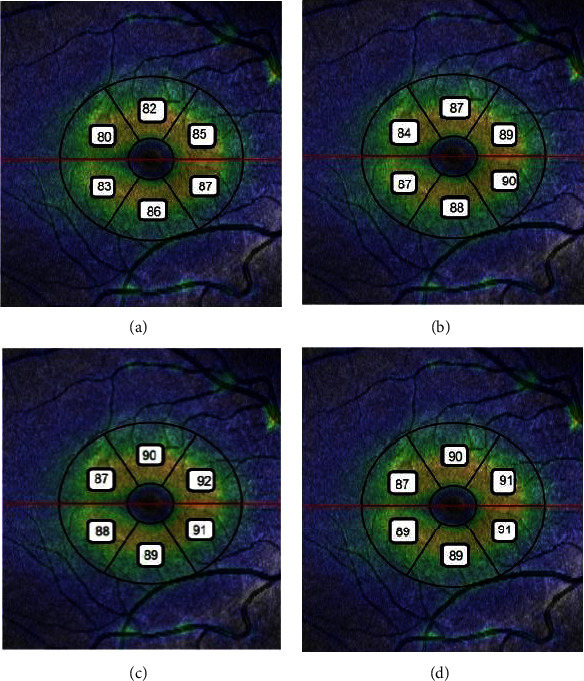
The mean GCL + IPL thickness (*μ*m) of six sectors in four studied group: (a) treated ROP; (b) regressed ROP; (c) preterm without ROP; (d) full-term children.

The post hoc analysis of GCL + IPL thickness profile is demonstrated in Table 4. The superior sector of GCL + IPL thickness in the treated group was significantly thinner than both control and preterm groups (*p*=0.012 and *p*=0.006, respectively). It was also thinner in the superotemporal sector in the treated group than both control and preterm groups (*p*=0.018 and *p*=0.016, respectively). In the inferotemporal sector the GCL + IPL was thinner in the treated group than both control and preterm groups (*p*=0.018 and *p*=0.005, respectively). The GCL + IPL in the superonasal sector were significantly thinner in treated group than preterm children (*p*=0.018). However the inferior and inferonasal sectors were not significantly different between groups (*p*=0.46 and *p*=0.19, respectively) and there was no significant difference in GCL + IPL thickness between treated and regressed groups in any sector (*P* > 0.05) (Figure 1).

## 4. Discussion

This study evaluated biometric and OCT parameters in children aged 4–8 years diagnosed with ROP and previous IVB treatment with three other age and gender-matched groups, including regressed ROP children without treatment, preterm children without a history of ROP, and full-term children. Children with a history of ROP and IVB treatment in our study achieved favorable visual and refractive outcomes with no significant differences found between BCVA and SE between groups.

In agreement with previous reports, our data show shorter AL in ROP children than in controls [4, 14]. Prousali et al. reported shorter AL as measured using the IOL Master in children aged 6–8 with regressed ROP compared to the control group [14]. Fieß et al. [4], in a Scheimpflug imaging study of children aged 4–10 years with a history of ROP, showed a decrease in AL in preterm children aged ≤7 compared to full-term. They hypothesized this reduction in AL was “a result of incomplete postnatal development until the age of 8 years.” By contrast, Wu et al. [13] reported no difference in AL between children with a history of treated ROP using cryotherapy or laser photocoagulation than full-term children aged 6–14 years using contact biometry. This study reports on the outcomes of children with a history of ROP treated with IVB. The mean of AL of the IVB-treated group in this study was 22.05 ± 1.03 mm which is in keeping with the IVB-treated group in the recent longitudinal study by Lee et al. [15] (22.2 ± 0.8 mm) in 4–6 year-old children. Factors such as various measurement techniques (contact vs. noncontact biometry), different age ranges, lack of a control group, and various treatment modalities may account for this variation in AL differences reported in the aforementioned studies.

The cornea was steeper in the ROP groups and the white-to-white diameter was smaller in all three preterm children, irrespective of ROP history. These findings support a recently published study by Kumarakulasinghe et al. [16], which showed preterm children without a history of ROP aged 7–9 years, had a smaller white-to-white diameter than term children. The smaller cornea in preterm children may be attributed to the arrested normal corneal growth, which is mainly reported to occur intrauterine [17].

The SE in the treated ROP group was comparable with other groups. Lu et al. [11] studied 3–8 years-old children who were treated with laser or IVB and reported that the mean SE in the IVB group was −0.53 ± 3.12 D which is similar to the results of this study (−0.29 ± 2.55 D). Anti-VEGF injection has been shown to promote regression of ROP and results in normal retinal vascularization [2], and better refractive and developmental outcomes than laser photocoagulation [3, 7]. The increase in myopia in laser-treated eyes is postulated to be due to an increase in LT and posterior lens curvature and a shallow ACD [11]. No significant difference was observed in LT, ACD, and CCT in treated versus other groups in this study.

Choroidal thickness was comparable between all groups in this study. In a study of children aged 5–10 years with or without a history of regressed ROP, no significant difference was found between choroidal thickness between preterm and control children [18]. Fieß et al. [19] also found no difference in choroidal thickness of preterm children aged 4–10 years regardless of the presence of ROP, compared to control with three infants undergoing laser photocoagulation treatment study. In contrast, several previous studies report a thinner choroid in preterm children [20–22]. In an animal model, postnatal hyperoxia-induced choroidal involvement played a vital key role in ROP's pathogenesis [23]. The difference in the results of this study compared to the above might be explained by several factors, including systemic and ocular variables affecting choroidal thickness, the nonhomogenous grouping in the studies, different OCT devices utilised, and different treatment modalities. Future studies should investigate other markers, such as choroidal vascularity index (CVI), which has been suggested to be a better parameter than choroidal thickness for evaluating choroidal disorders [19, 24].

All three preterm groups had thicker central macular thickness than the control, while treated ROP children had a significantly thicker macula than preterm children in this study. A thicker macula in preterm children has been reported in several previous studies [25, 26]. The increase in foveal thickness in preterm infants, especially those with ROP, has been suggested to be due to a failure of lateral cell migration of the inner retinal layers away from the fovea [20, 27]. Although in a study by Lee et al. [28], the laser destruction effect on retinal tissue was suggested to result in a halt in cellular migration during foveal development compared to IVB-treated eyes, a recent longitudinal study showed the effect of GA in foveal thickness was much more significant than the different treatment modalities in ROP with no significant difference in macular thickness observed after adjusting for GA [15]. The lower GA was associated with a thicker macula in several studies and is in keeping with the results of this study [19, 25]. Visual acuity was not correlated with macular thickness in this study, and similar results have been reported in previous investigations [13, 25, 27, 29]. While this has been suggested to be due to the independence of inner retinal migration to photoreceptor maturation [27], other studies have reported an association between visual acuity and fovea thickness [15, 19].

GCL + IPL was thinner in all sectors except for inferior and inferonasal in the treated group than in both preterm and full-term children, which is in keeping with several previous studies [19, 30]. The neuronal damage related to ROP or laser photocoagulation has been suggested for thinning of GCL in children with a history of ROP [28]. Fieß et al. [19] found thinner GCL + IPL in preterm children with GA ≤28 weeks, but no significant difference was observed in those with GA 29–32 weeks. However, the findings of GCL thickness in preterm infants are inconsistent with Yanni et al. [29] and Rosén et al. [5] in extremely preterm children (<27 weeks of GA), reporting increased GCL + IPL thickness compared to controls. Furthermore, in another study, Park et al. [20] reported no significant difference between preterm and full-term children with a mean age of 6 years in GCL + IPL thickness. Methodological differences in studies, including OCT devices, small sample sizes in subgroups of ROP, different age ranges, history of ROP treatment, or perinatal characteristics, are among the reasons for the observed difference in findings.

IOP was significantly higher only in the regressed ROP group compared to the preterm group in this study (*P*=0.012). In a recent study of preterm children without ROP, no difference in IOP as measured by the Applanation Tonometer (Tono-Pen XL, Reichert, Depew, NY) was observed compared to full-term children [16]. IOP was measured with IC200 rebound tonometry in the present study, which has previously been reported to have good agreement with standard applanation tonometry [31]. Rebound tonometry is also thought to provide more objective IOP measurements in pediatric patients, as it does not require topical anesthesia.

This study included a relatively large sample size in each subgroup and simultaneously evaluated OCT and biometrical parameters in children with a history of ROP, including those with prior IVB treatment. This study, however, has some limitations, including a single-center, cross-sectional design, which may have reduced the representativeness of our cohort. Second, the enrollment of children with good cooperation may have introduced an element of bias.

In conclusion, shorter AL, steeper cornea, and a negative correlation between GA and macular thickness were observed in treated and regressed ROP children whilst there was no significant difference noted in SE or refractive cylinder between the groups. This study documents the change in refractive error as well as macular abnormalities, and biometric differences in children with ROP with or without prior treatment and provides valuable information for healthcare providers and funders planning screening and follow-up of these children, particularly during preschool years and during the time period where these children are particularly prone to developing amblyopia. Future studies with larger cohorts are required to directly investigate the effect of various ROP treatments on biometric and OCT characteristics of children and their final impact on visual acuity.

## Figures and Tables

**Table 1:** Demographic data of the study population (*n*: 30 each group).

Parameters	Group 1	Group 2	Group 3	Group 4	*P*
GA, weeks median (IQR)	28 (1.5)	30 (3.5)	32 (3)	37	<0.001^∗^
BW, g median (IQR)	1045 (342.5)	1360 (363.75)	1625 (325)	3100 (650)	<0.001^∗^
Delivery, natural *n* (%)	11 (36.7)	9 (30)	6 (20)	17 (56.7)	0.02^∗^^a^
UCVA, logMAR mean ± SD	0.2 ± 0.37	0.02 ± 0.06	0.06 ± 0.12	0.1 ± 0.26	0.08
BCVA, logMAR mean ± SD	0.05 ± 0.09	0.01 ± 0.03	0.02 ± 0.06	0.01 ± 0.03	0.05
ROP zone no (%)					
I	17 (56.7)	0 (0)	N/A	N/A	<0.001^∗^
II	13 (43.3)	24 (80)	N/A	N/A	
III	0 (0)	6 (20)	N/A	N/A	
ROP stage no (%)					
1	1 (3.3)	4 (13.3)	N/A	N/A	<0.001^∗^
2	4 (13.3)	26 (86.7)	N/A	N/A	
3	25 (83.3)	0 (0)	N/A	N/A	
SE, D, mean ± SD	−0.29 ± 2.55	0.56 ± 0.60	0.76 ± 1.10	0.24 ± 1.64	0.3
IOP (mm Hg)	15.85 (4.65)	16.95 (5.25)	15.40 (2.05)	16 (2.15)	0.012^∗^

Group 1: treated ROP; Group 2: regressed ROP; Group 3: preterm without ROP; Group 4: full-term. Values are reported as mean ± standard deviation or median (interquartile range). GA, gestational age; IQR, interquartile range; BW, birth weight; BCVA, best-corrected visual acuity; UCVA, uncorrected visual acuity; logMAR, logarithm of the minimum angle of resolution; SD, standard deviation; D, diopters; N/A, not applicable; SE, spherical equivalent; IOP, intraocular pressure. ^∗^Statistically significant result. All *P* values were obtained using Kruskal–Wallis test except indicated ^a^using chi-square test.

**Table 2:** Biometric data parameters of the study population (*n*: 30 each group).

Parameters	Group 1	Group 2	Group 3	Group 4	*P*
AL (mm)	21.855 (1.27)	21.925 (1.43)	22.535 (0.91)	22.785 (1.25)	0.001^∗^^a^
					1, 4 *p*=0.002; 2, 4 *p*=0.002^b^

ACD (mm)	3.354 ± 0.29	3.35 ± 0.22	3.35 ± 0.27	3.53 ± 0.26	0.02^∗^
					3, 4 *p*=0.045

LT (mm)	3.62 ± 0.17	3.56 ± 0.18	3.51 ± 0.15	3.52 ± 0.17	0.05

CCT (*μ*m)	524 (66)	556 (59)	532 (30.75)	539.500 (31.50)	0.12^a^

K1 (D)	45.61 ± 1.55	44.76 ± 1.95	43.67 ± 1.44	42.94 ± 1.37	<0.001^∗^
					1, 3 *p* < 0.001; 1, 4 *p* < 0.001
					2, 3 *p*=0.047; 2, 4 *p* < 0.001

K2 (D)	46.59 ± 1.58	45.75 ± 1.90	44.96 ± 1.81	44.17 ± 1.43	<0.001^∗^
					1, 3 *p*=0.002; 1, 4 *p* < 0.001
					2, 4 *p*=0.003

Mean K (D)	46.10 ± 1.54	45.24 ± 1.91	44.30 ± 1.57	43.54 ± 1.36	<0.001^∗^
					1, 4 *p* < 0.001; 2, 4 *p* < 0.001

Cyl (D)	−0.97 ± 0.5	−0.99 ± 0.56	−1.29 ± 088	−1.23 ± 0.68	0.62

WTW (mm)	11.63 ± 0.50	11.67 ± 0.49	11.92 ± 0.41	12.28 ± 0.45	<0.001^∗^
					1, 4 *p* < 0.001; 2, 4 *p* < 0.001
					3, 4 *p*=0.015

Group 1: treated ROP; Group 2: regressed ROP; Group 3: preterm without ROP; Group 4: full-term. Data are mean ± standard deviation or median (interquartile range). AL, axial length; ACD, anterior chamber diameter; LT, lens thickness; CCT, central corneal thickness; K1, flattest keratometry; K2, steepest keratometry; D, diopter; Cyl, cylinder; WTW, white-to-white. ^∗^Statistically significant result. All *P* values were obtained using ANOVA test except indicated. ^a^using Kruskal–Wallis. ^b^Pairwise comparison between groups.

**Table 3:** Macular thickness of the nine early treatment diabetic retinopathy study regions (*n*: 30 each group).

Parameters	Group 1	Group 2	Group 3	Group 4	*P*
Central (*µ*m)^a^	249.86 ± 34.46	237.23 ± 21.87	228.60 ± 18.92	211.63 ± 15.99	<0.001^∗^
					1, 3 *p*=0.004; 1, 4 *p* < 0.001
					2, 4 *p* < 0.001; 3, 4 *p*=0.034

Inner superior (*µ*m)^b^	295.03 ± 15.45	303.83 ± 15.61	305 ± 12.52	304.20 ± 12.47	0.02^∗^
					1,4 *p*=0.034

Inner inferior (*µ*m)	298 (15.25)	299.50 (22)	299 (21)	301.50 (14.5)	0.97^e^

Inner nasal (*µ*m)	296.36 ± 15.47	300.90 ± 13.25	302.43 ± 13.35	298.80 ± 13.66	0.36

Inner temporal (*µ*m)	285.33 ± 16.69	292.53 ± 12.84	289.46 ± 14.68	288.86 ± 11.79	0.27

Outer superior (*μ*m)^c^	267.50 (15.25)	273 (13.5)	274.50 (14.25)	274.50 (21.5)	0.16^e^

Outer inferior (*µ*m)	265.93 ± 18.79	269.20 ± 12.63	269.73 ± 13.70	268.06 ± 15.18	0.77

Outer nasal (*µ*m)	284.83 ± 19.03	287.83 ± 12.29	290.43 ± 11.43	289.20 ± 14.45	0.48

Outer temporal (*µ*m)	259 (17.25)	260.50 (16)	258.50 (12.25)	254.50 (21.5)	0.76^e^

Choroidal thickness (*µ*m)	302.40 ± 77.51	294.83 ± 54.78	290.43 ± 55.26	292.40 ± 72.55	0.90

Group 1: treated ROP; Group 2: regressed ROP; Group 3: preterm without ROP; Group 4: full-term. Data are mean ± standard deviation or median (interquartile range). ^∗^Statistically significant result. ^a^Central macular thickness within 1 mm diameter. ^b^Inner areas of macular thickness within 3 mm diameter. ^c^Outer areas of macular thickness within 6 mm diameter. ^d^Pairwise comparison between groups. ^e^*P* values were obtained using Kruskal–Wallis.

**Table 4:** GCL + IPL thickness profile (*n*: 30 each group).

Sectors	Group 1	Group 2	Group 3	Group 4	*P*
Superior(*μ*m)	85.50 (11.5)	88.50 (7.25)	90.50(8.25)	90.50 (10.25)	0.003^∗^
					1, 3 *p*=0.006; 1, 4 *p*=0.012^a^

Inferior (*μ*m)	88.50 (7)	89 (8.75)	90.50 (6.50)	90 (7.5)	0.46

Superonasal (*μ*m)	88 (11.5)	90.50 (8.5)	91.50 (6.25)	93 (9)	0.01^∗^
					1, 3 *p*=0.018

Inferonasal (*μ*m)	89.50 (6.25)	91(6.5)	91 (7.75)	93 (9.25)	0.19

Superotemporal (*μ*m)	83.50 (9)	85.50 (6.75)	86 (8.5)	86.50 (7.75)	0.005^∗^
					1, 3 *p*=0.016; 1, 4 *p*=0.018

Inferotemporal (*μ*m)	84.50 (8.50)	89 (7.75)	88.50 (9.25)	90 (6.75)	0.006^∗^
					1, 3 *p*=0.005; 1, 4 *p*=0.018

Group 1: treated ROP; Group 2: regressed ROP; Group 3 preterm without ROP; Group 4: full-term. Data are median (interquartile range). ^∗^Statistically significant result. All *P* values were obtained using Kruskal–Wallis test. ^a^Pairwise comparison between groups.

## Data Availability

The data that support the findings of this study are available from the corresponding author upon reasonable request.
